# Nodulation Experiment by Cross-Inoculation of Nitrogen-Fixing Bacteria Isolated from Root Nodules of Several Leguminous Plants

**DOI:** 10.4014/jmb.2310.10025

**Published:** 2023-12-30

**Authors:** Ahyeon Cho, Alpana Joshi, Hor-Gil Hur, Ji-Hoon Lee

**Affiliations:** 1 Department of Agricultural Chemistry, Jeonbuk National University, Jeonju 54896, Republic of Korea; 2 Department of Bioenvironmental Chemistry, Jeonbuk National University, Jeonju 54896, Republic of Korea; 3 Department of Agriculture Technology & Agri-Informatics, Shobhit Institute of Engineering & Technology, Meerut 250110, India; 4 School of Earth Sciences and Environmental Engineering, Gwangju Institute of Science and Technology, Gwangju 61005, Republic of Korea

**Keywords:** Biological nitrogen fixation, indole acetic acid, nitrogen-fixing bacteria, phylogenetic, root nodulation

## Abstract

Root-nodule nitrogen-fixing bacteria are known for being specific to particular legumes. This study isolated the endophytic root-nodule bacteria from the nodules of legumes and examined them to determine whether they could be used to promote the formation of nodules in other legumes. Forty-six isolates were collected from five leguminous plants and screened for housekeeping (16S rRNA), nitrogen fixation (*nifH*), and nodulation (*nodC*) genes. Based on the 16S rRNA gene sequencing and phylogenetic analysis, the bacterial isolates WC15, WC16, WC24, and GM5 were identified as *Rhizobium*, *Sphingomonas*, *Methylobacterium*, and *Bradyrhizobium*, respectively. The four isolates were found to have the *nifH* gene, and the study confirmed that one isolate (GM5) had both the *nifH* and *nodC* genes. The Salkowski method was used to measure the isolated bacteria for their capacity to produce phytohormone indole acetic acid (IAA). Additional experiments were performed to examine the effect of the isolated bacteria on root morphology and nodulation. Among the four tested isolates, both WC24 and GM5 induced nodulation in *Glycine max*. The gene expression studies revealed that GM5 had a higher expression of the *nifH* gene. The existence and expression of the nitrogen-fixing genes implied that the tested strain had the ability to fix the atmospheric nitrogen. These findings demonstrated that a nitrogen-fixing bacterium, *Methylobacterium* (WC24), isolated from a *Trifolium repens*, induced the formation of root nodules in non-host leguminous plants (*Glycine max*). This suggested the potential application of these rhizobia as biofertilizer. Further studies are required to verify the N_2_-fixing efficiency of the isolates.

## Introduction

The global food production supply is under increasingly severe stress due to the expanding human population and adverse environmental conditions. While synthetic nitrogen fertilizers have addressed those problems by providing a solution for efficient crop production and contributing to the growing world's food supply, the excessive application of chemical fertilizer is one of the leading causes of pollution of groundwater. Polluted groundwater is an increasing hazard to the health of both humans and the environment [1]. Environmentally friendly alternative fertilizers are crucial for the sustainability of agriculture. Biological nitrogen fixation (BNF) offers a sustainable and cost-effective alternative to chemical fertilizer for use on legumes. BNF improves the soil fertility by fixing the atmospheric nitrogen (N_2_) into biologically available ammonium, which the plants then utilize to synthesize various biomolecules. This process is performed exclusively by prokaryotes, including archaea and bacteria [2]. BNF accounts for 65% of the nitrogen utilized for the effective production of crops, which demonstrates the economic importance of rhizobia in agriculture [3].

The '*nod*' strategy is a mechanism to induce root nodule formation in legumes [4, 5]. Legumes have an exceptional ability to develop a symbiotic relationship with rhizobia. The symbiotic association between legumes and rhizobia is highly specific, and each rhizobial species interacts only with particular legumes and vice versa. Rhizobia such as *Rhizobium*, *Mesorhizobium*, *Bradyrhizobium*, *Azorhizobium*, *Allorhizobium*, and *Sinorhizobium* establish symbiotic associations by chemotactically responding to flavonoid molecules which are released by the host legumes as signals. These flavonoid compounds induce the expression of nodulation genes (*nod*) in the rhizobia, which in turn produce strain-specific lipo-chito-oligosaccharide (LCO) signal molecules (Nod factors). These then trigger mitotic cell division in the roots, leading to nodule formation [6-8]. The *nodC* gene encodes N-acetyl glucosaminyl transferase and polymerizes UDP-N-accetyl-D-glucosamine into the chitin-tetraose or chitin-pentaose. The *nodC* is commonly used as a nodulation gene marker involved in the Nod factor assembly, and provides for the host specificity. Biological nitrogen fixation is mediated by the nitrogenase enzyme complex known as *nif* regulon. The *nifH* gene (nitrogen fixation gene H) is a member of the *nif* regulon that encodes dinitrogenase reductase. It is a genetically conserved marker gene that identifies the nitrogen-fixing in bacteria and archaea [9, 10]. The symbiotic microorganisms fix the nitrogen inside the nodules in the form of bacteroids, known as symbiosomes using the catalytic nitrogenase enzymes. They do this in the presence of leghemoglobin (symbiotic hemoglobin), a hemeprotein produced due to the symbiotic association between the bacteroids and legumes [11, 12].

This legume-rhizobium symbiosis model has been a substantive area for research focus as legumes are important food and economic crops [13-16]. BNF is a cost-effective and sustainable method for effective crop production, and for maintaining long-term crop productivity. It is essential to identify the best-performing nitrogen-fixing bacterial symbionts in the legumes-rhizobia symbioses for the most efficient BNF, and to use these to develop biofertilizers [17]. In addition to N_2_ fixation, rhizobia have several other beneficial impacts on plant growth, including synthesizing the various metabolites and enzymes during nodule formation. Rhizobia produce a variety of phytohormones, such as auxins (indole acetic acid), cytokinins, and gibberellic acid (GA). Indole acetic acid (IAA) is a phytohormone that regulates the various physiological processes of plants, such as cell division, growth, and tissue differentiation [18-21].

This study isolated the rhizobia strains from root nodules of several leguminous plants growing in different locations. These isolates were identified with phylogenetic analysis based on 16S rRNA gene sequences. The nitrogenase gene (*nifH*) and the nod factor gene (*nodC*) were identified and characterized by amplification from the isolated bacterial strains using PCR. The expression levels of the *nifH* gene were quantified using qRT-PCR. Although it is well known that the legume-rhizobium symbiosis is host-specific, the nodulation capacity of the isolated rhizobia strains was tested in different legumes using a cross-inoculation technique.

## Materials and Methods

### Plant Sampling and Nodule Collection

Five different leguminous plants were sampled from local and agricultural areas in Jeollabuk-do, South Korea, collecting them for their root nodules (Table 1). The plants were: *Trifolium repens* (white clover), *Wisteria floribunda* (Japanese wisteria), *Pisum sativum* (pea), *Vigna radiata* (mung bean), and *Glycine max* (soybean). From the sampled plants, the root nodules were collected within 1-3 days after sampling and were stored at 4ºC until the experiments were conducted. All of the shovels and tweezers were sterilized with an autoclave before the plant sampling and before collecting root nodules.

### Isolation of Endophytic Bacteria from the Root Nodules of the Legumes

All of the collected nodules were surface-sterilized by treating them with 95% ethanol and 5% sodium hypochlorite solution for 5 min, followed by rinsing three times with sterile water. Five samples of the surface-sterilized nodules were crushed in a physiological saline water (0.85% NaCl) with sterilized tweezers. The crushed nodule solutions were diluted up to ×10^-3^, and 40 μl of each solution was spread onto four types of agar media: Tryptic soy broth (TSB), Reasoner's 2A agar (R2A), yeast extract mannitol (YEM), and glucose peptone (GP). The cultures were incubated at 30°C.

### DNA Extraction and Sequencing

Genomic DNA was extracted using an Inclone Genomic Plus DNA Prep Kit (Intron Biotechnology, Republic of Korea) and purified with an AccuPrep PCR/Gel Purification Kit (Bioneer, Republic of Korea). PCR was performed in 20 μl of the reaction solution, which contained nuclease-free water, 1.0 μl of DNA template (10 ng/μl), AccuPower Taq PCR PreMix (Bioneer), and 1.0 μl of each primer (10 μM). All of the primer sets for each PCR are summarized in Table 2. The PCR amplification of the 16S rRNA gene was performed using the bacterial universal 27F [22] and 1492R [23] primers with an initial denaturation at 94°C for 2 min, followed by 30 cycles of denaturation at 94°C for 30 s, annealing at 50°C for 30 s, and extension at 72°C for 90 s. The final extension was at 72°C for 5 min. The thermal profile used to amplify the *nifH* gene, which used the Pol primer set [24], included the following: pre-denaturation at 95°C for 5 min, followed by 30 cycles of denaturation at 94°C for 1 min, annealing at 58°C for 1 min, and extension at 72°C for 1 min, and a final extension at 72°C for 5 min. The thermal profiles using IGK3 and DVV primers [10] were initial denaturation at 95°C for 5 min, followed by 30 cycles of denaturation at 94°C for 30 s, annealing at 58°C for 1 min, and extension at 72°C for 30 s, with a final extension at 72°C for 7 min. The amplification of the *nodC* gene was performed using the specific primer set of *NodC*_F 540 and *NodC*_R 1160 [25]; with initial denaturation at 94°C for 3 min, followed by 30 cycles of denaturation at 94°C for 45 s, annealing at 50°C for 30 s, and extension at 72°C for 1 min, and a final extension at 72°C for 5 min. Agarose gel electrophoresis (1.5%) was used to visualize the amplified PCR product. The PCR products were purified using an AccuPrep PCR/Gel Purification Kit, and were sequenced on an ABI3730 using BigDye Terminator v3.1 (Thermo Fisher Scientific, USA).

### Sequences and Phylogenetic Analyses

The sequences were assembled and edited manually using the BioEdit v7.2.5. The consensus sequences of each gene were submitted to the Basic Local Alignment Search Tool (BLAST) of the National Center for Biotechnology Information (NCBI) to assess for similarities. The aligned sequences were deposited in the NCBI GenBank and obtained the following accession numbers: OR553256 (16S rRNA), OR553281 (16S rRNA), OR553378 (16S rRNA), OR553398 (16S rRNA), OR584330 (*nodC*), OR584331 (*nifH*), OR584332 (*nifH*), OR584333 (*nifH*), and OR584334 (*nifH*). Phylogenetic analyses of the 16S rRNA, *nifH*, and *nodC* genes were conducted using the Neighbor-joining method with 10,000 bootstraps in MEGA11 [26-27].

### Symbiotic Properties

The three types of soybean seeds used in the plant experiment were: *Trifolium repens*, *Glycine max*, and *Phaseolus vulgaris*. All of the seeds used were surface-sterilized with a 2% sodium hypochlorite solution (2 min) and 70% ethanol (1 min), and then rinsed several times with sterile distilled water. The seeds were germinated in 1% water-agar media under dark conditions at 28°C for 2-3 days, and the treated seeds were then planted in pots filled with vermiculite (with one seed per pot). Two samples were used as the controls: one was inoculated with *Shewanella oneidensis* MR-1 [28], and the other was not inoculated with any microbial strains. All of the selected strains and controls were grown in an R2A liquid medium and inoculated in the soil, with as much as 3×10^8^ cells, using a syringe. The plants were grown for 40 days under controlled conditions in the incubator at 28°C, with 16 h for light and 8 h for dark conditions. The plants were harvested 40 days after seeding and were checked for whether root nodules had formed. They were then dried in an oven at 70°C. The dry weight of the roots and root nodules were recorded. All of the experiments were conducted in triplicate.

### Indole Acetic Acid (IAA) Production

The Salkowski method was used in order to determine whether the isolated strain had produced an indole compound [29]. All of the strains used in the experiment were cultured in an R2A liquid medium containing L-tryptophan (0.5 g/l), and were then incubated for seven days at 28°C at 90 rpm. The two control samples were used as negative controls: one was inoculated with *S. oneidensis* MR-1, and the other control was not inoculated with any bacterium. After 7 days, the culture solution was centrifuged at 10,000 rpm for 1 min to collect the supernatant and filtered using a 0.45 μl filter with the syringe. One ml of the supernatant was mixed with 2 ml of Salkowski reagent (which was dissolved in 4.5 g of iron chloride in 1 L of 10.8 M sulfuric acid), and was then incubated at room temperature for 30 min. A color change to pink indicated the production of indole. IAA production was quantified from the supernatant absorbance using a spectrophotometer with a wavelength of 530 nm. The experiment was conducted in triplicates, and the average was used to calculate the final concentration (μM) by substituting it into the calibration curve. The relative fold increase in IAA production was calculated based on the control value.

### Quantitative Reverse Transcription PCR (qRT-PCR) Analysis of *nifH* Gene

The expression levels of the *nifH* gene were determined by quantitative reverse transcription PCR (qRT-PCT) analysis. Bacterial RNAs were extracted from the root nodules of the *Glycine max* that was inoculated with the strains of GM5 and WC24, using and RNeasy Plant Mini Kit (Qiagen, Germany). The liquid nitrogen-frozen nodules were crushed in the homogenizer to extract the RNA. A first strand cDNA synthesis was carried out by the PrimeScript 1st strand cDNA Synthesis Kit (Takara Bio Inc., Japan) following the manufacturer's instructions. The qRT-PCR was performed on a CFX Connect Real-Time System (Bio-Rad Laboratories, Inc., USA) using the AccuPower GreenStar RT-qPCR Premix (Bioneer), and primer sets of Pol [24] and IGK3+DVV [10] for GM5 and WC24 strains, respectively. The primer sequences are given in Table 2. The expression levels of the *nifH* were determined and compared to the housekeeping gene GAPDH [30].

## Results

### Isolation and Amplification of the Housekeeping (16S rRNA) and Symbiotic (*nifH* and *nodC*) Genes

In this study, 46 strains were isolated from plant root nodules collected from *Trifolium repens* (WC), *Wisteria floribunda* (WF), *Pisum sativum* (PS), *Vigna radiate* (ND), and *Glycine max* (GM). High-quality genomic DNA was procured from each isolate and amplified using gene-specific primers for the 16S rRNA, *nifH*, and *nodC* genes. Among the 46 isolated strains, only four isolates - three from *Trifolium repens* (WC15, WC16, and WC24) and one from *Glycine max* (GM5) - were used. They were selected for their morphologies on agar plates and for the successfully acquired 16S rRNA gene sequences. The rest of the isolates showed morphological similarities or were unsuccessful in their sequencing of the 16S rRNA gene. The *nifH* gene was amplified using the IGK3/DVV and PolF/PolR primer pairs, and an amplicon of the desired length (300~400 bp) was produced. Only one isolate (GM5) produced the desired 600 bp fragment from the *nodC* gene using the *nodC*540F/1160R primer pair. The amplified products of the 16S rRNA, *nifH*, and *nodC* genes were sequenced and compared using the NCBI BLAST tool. Based on 16S rRNA gene sequences, four out of 46 tested isolates - WC15, WC16, WC24, and GM5 - were identified as *Rhizobium* sp., *Sphingomonas* sp., *Methylobacterium* sp., and *Bradyrhizobium* sp., respectively. The top three BLASTn scores of each gene sequence are presented in Table 3.

### Phylogenetic Analysis of the Housekeeping (16S rRNA) and Symbiotic (*nifH* and *nodC*) Genes

**Fig. 1:**
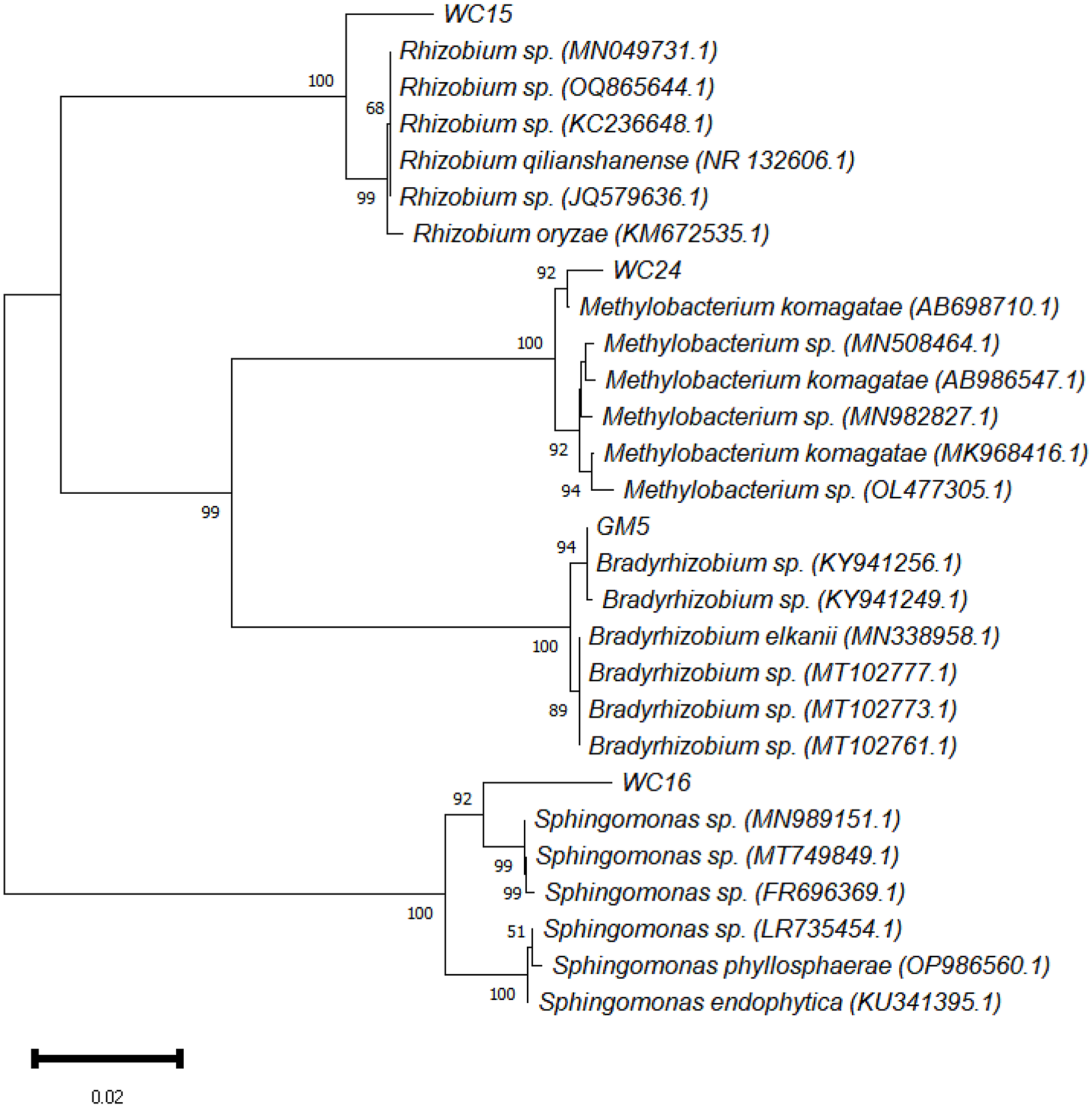
Phylogenetic tree of nitrogen-fixing bacteria (WC15, WC16, WC24, and GM5) isolated from leguminous root nodules based on 16S rRNA gene sequence. The number indicates the levels of bootstrap support based on 10,000 replicates.

Phylogenetic analysis of the isolated strains was conducted based on the housekeeping (16S rRNA) and symbiotic (*nifH* and *nodC*) gene sequences. The 16S rRNA gene-based neighbor-joining tree analysis revealed the four isolates - WC15, WC16, WC24, and GM5 - exhibited the highest similarity to *Rhizobium*, *Sphingomonas*, *Methylobacterium*, and *Bradyrhizobium*, respectively (Fig. 1). Isolate WC15 shared a 98% sequence similarity with *Rhizobium* sp. (MN049731.1), *Rhizobium* sp. (OQ865644.1), *Rhizobium* sp. (KC236648.1), *Rhizobium qilianshanense* (NR_132606.1), *Rhizobium* sp. (JQ579636.1), and *Rhizobium oryzae* (KM672535.1) with 100% bootstrap support. Isolate WC16 shared a 99% sequence similarity with *Sphingomonas* sp. (MN989151.1), *Sphingomonas* sp.(MT749849.1), *Sphingomonas* sp. (FR696369.1), *Sphingomonas* sp. (LR735454.1), *Sphingomonas endophytica* (KU341395.1), and *Sphingomonas phyllosphaerae* (OP986560.1) with 100% bootstrap support. Isolate WC24 shared a 99% sequence similarity with *Methylobacterium komagatae* (AB698710.1), *Methylobacterium* sp.(MN508464.1), *Methylobacterium* sp. (MN982827.1), *M. komagatae* (MK968416.1), *M. komagatae* (AB986547.1), and *Methylobacterium* sp. (OL477305.1), with 100% bootstrap support. Isolate GM5 shared a 98% sequence similarity with *Bradyrhizobium* sp. (KY941256.1), *Bradyrhizobium* sp. (KY941249.1), *Bradyrhizobium elkanii* (MN338958.1), *Bradyrhizobium* sp. (MT102777.1), *Bradyrhizobium* sp. (MT102773.1), and *Bradyrhizobium* sp.(MT102761.1), with 100% bootstrap support.

**Fig. 2:**
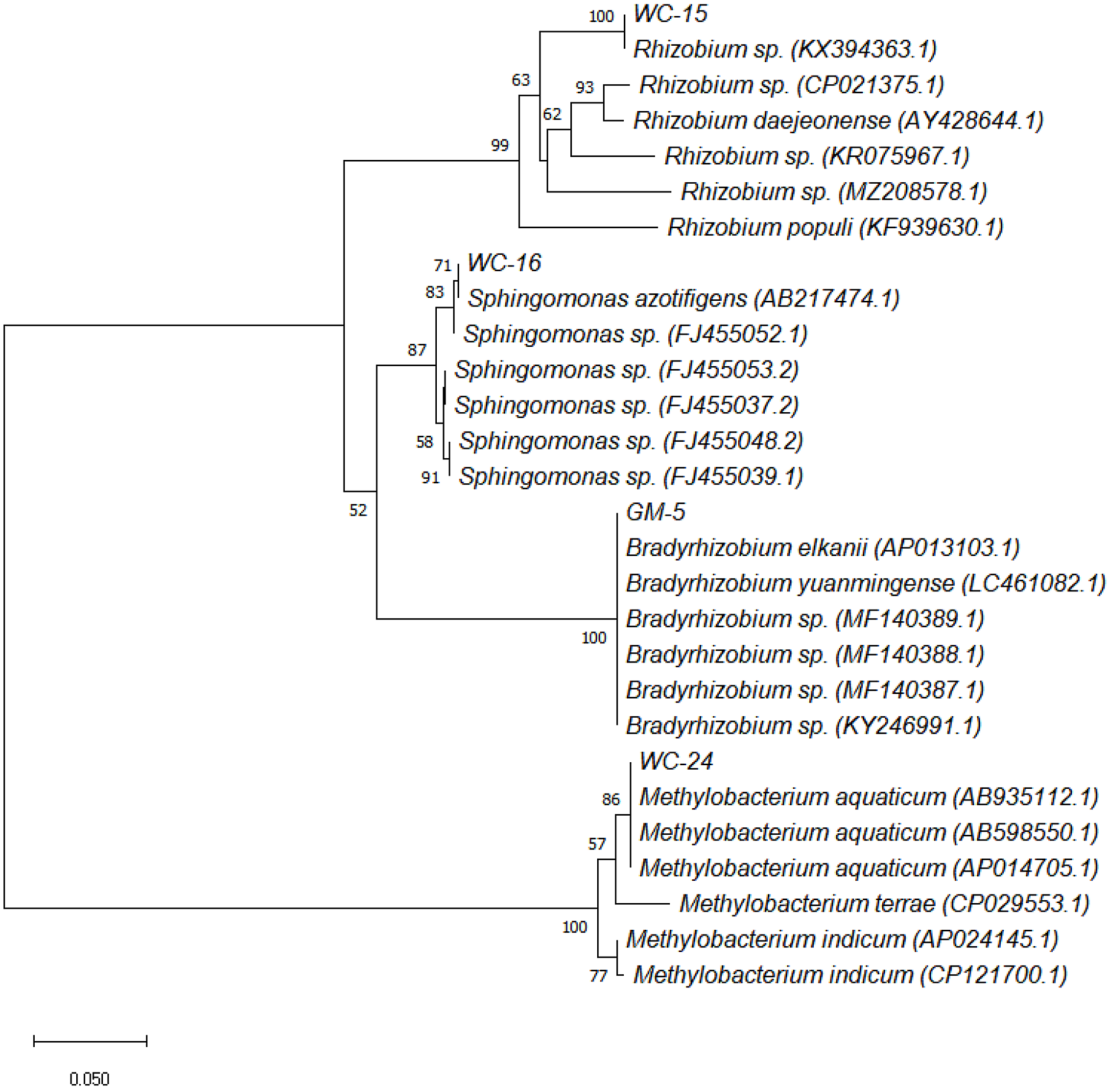
Phylogenetic tree of *nifH* gene from nitrogen-fixing bacterial isolates (WC15, WC16, WC24, and GM5). The number indicates the levels of bootstrap support based on 10,000 replicates.

Phylogenetic analysis based on the *nifH* gene grouped the test isolates (WC15, WC16, WC24, and GM5) into various clusters within the genus (Fig. 2). The *nifH* gene from WC15 displayed the highest similarities with those sequences from *Rhizobium* sp. (KX394363.1), *Rhizobium* sp. (CP021375.1), *Rhizobium* sp. (KR075967.1), *Rhizobium daejeonense* (AY428644.1), *Rhizobium populi* (KF939630.1), and *Rhizobium* sp. (MZ208578.1), with 99%bootstrap support. The *nifH* from WC16 displayed 98%-100% sequence similarity with *Sphingomonas azotifigens* (AB217474.1), *Sphingomonas* sp. (FJ455053.2), *Sphingomonas* sp. (FJ455037.2), *Sphingomonas* sp. (FJ455052.1), *Sphingomonas* sp. (FJ455048.2), and *Sphingomonas* sp. (FJ455039.1), with 88% bootstrap support. The *nifH* from WC24 exhibited 100% sequence similarities with *Methylobacterium aquaticum* (AB935112.1), *M. aquaticum* (AB598550.1), *M. aquaticum* (AP014705.1), *Methylobacterium indicum* (AP024145.1), *Methylobacterium terrae* (CP029553.1), and *M. indicum* (CP121700.1), with 100% bootstrap support. The *nifH* from GM5 showed 100%sequence similarities with *Bradyrhizobium elkanii* (AP013103.1), *Bradyrhizobium yuanmingense* (LC461082.1), *Bradyrhizobium* sp. (MF140389.1), *Bradyrhizobium* sp. (MF140388.1), *Bradyrhizobium* sp. (MF140387.1), and *Bradyrhizobium* sp. (KY246991.1), with 100% bootstrap support.

**Fig. 3:**
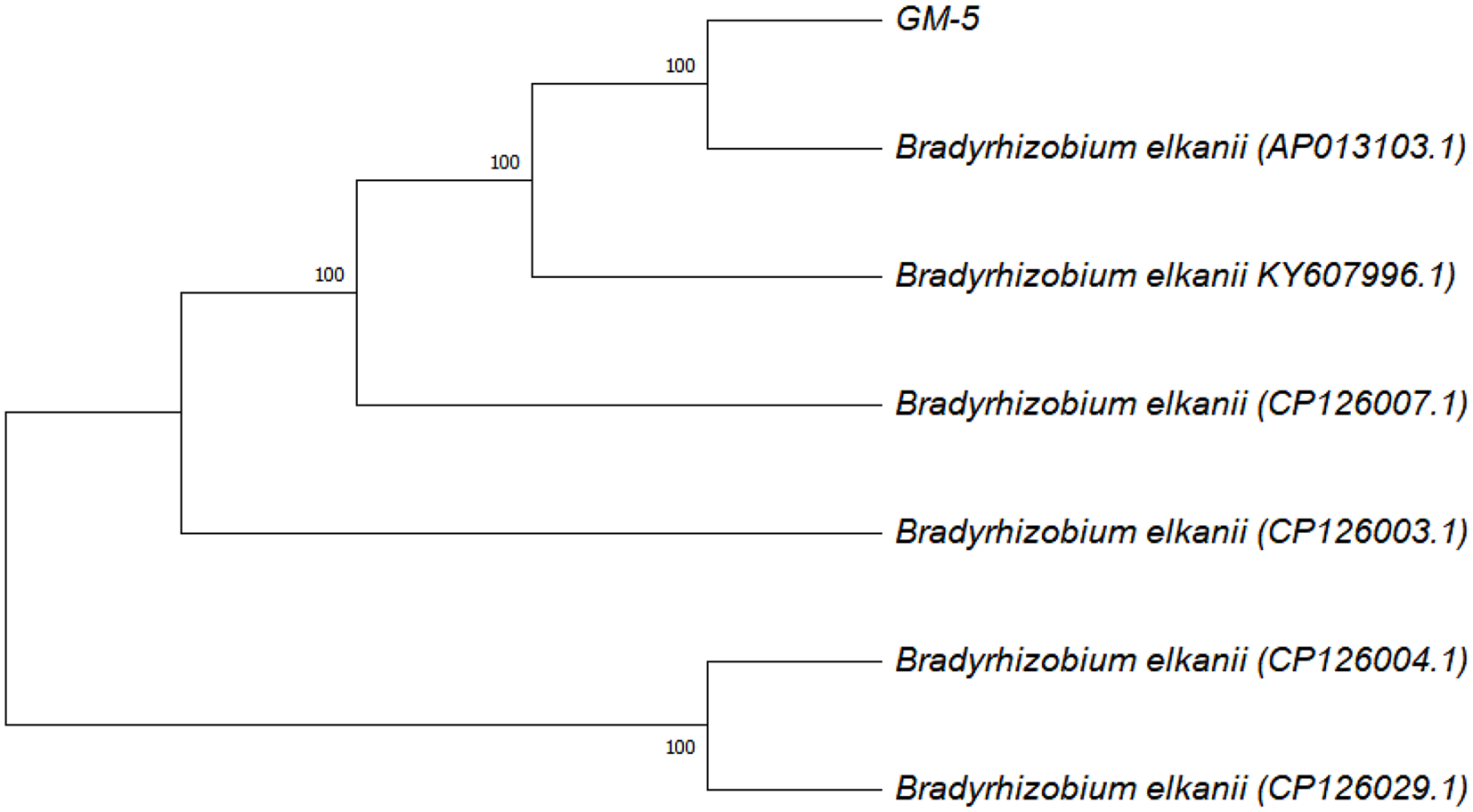
Phylogenetic tree of *nodC* gene from a nitrogen-fixing bacterium (GM5). The number indicates the levels of bootstrap support based on 10,000 replicates.

Phylogenetic analysis, based on the *nodC* gene, grouped the GM5 isolate with the genus *Bradyrhizobium* (Fig. 3). The isolate GM5 shared a 100% sequence similarity of the *nodC* gene with six different accessions from *B. elkanii* (AP013103.1, KY607996.1, CP126007.1, CP126003.1, CP126004.1, and CP126029.1), with 100%bootstrap support. Based on the *nifH* and *nodC* genes, the neighbor-joining phylogenies demonstrated that the tested isolates were consistently grouped in the housekeeping (16S RNA) and symbiotic (*nifH* and *nodC*) genes’ phylogenies.

### Detection of the Indole Acetic Acid (IAA) Production in Bacterial Isolates

**Fig. 4:**
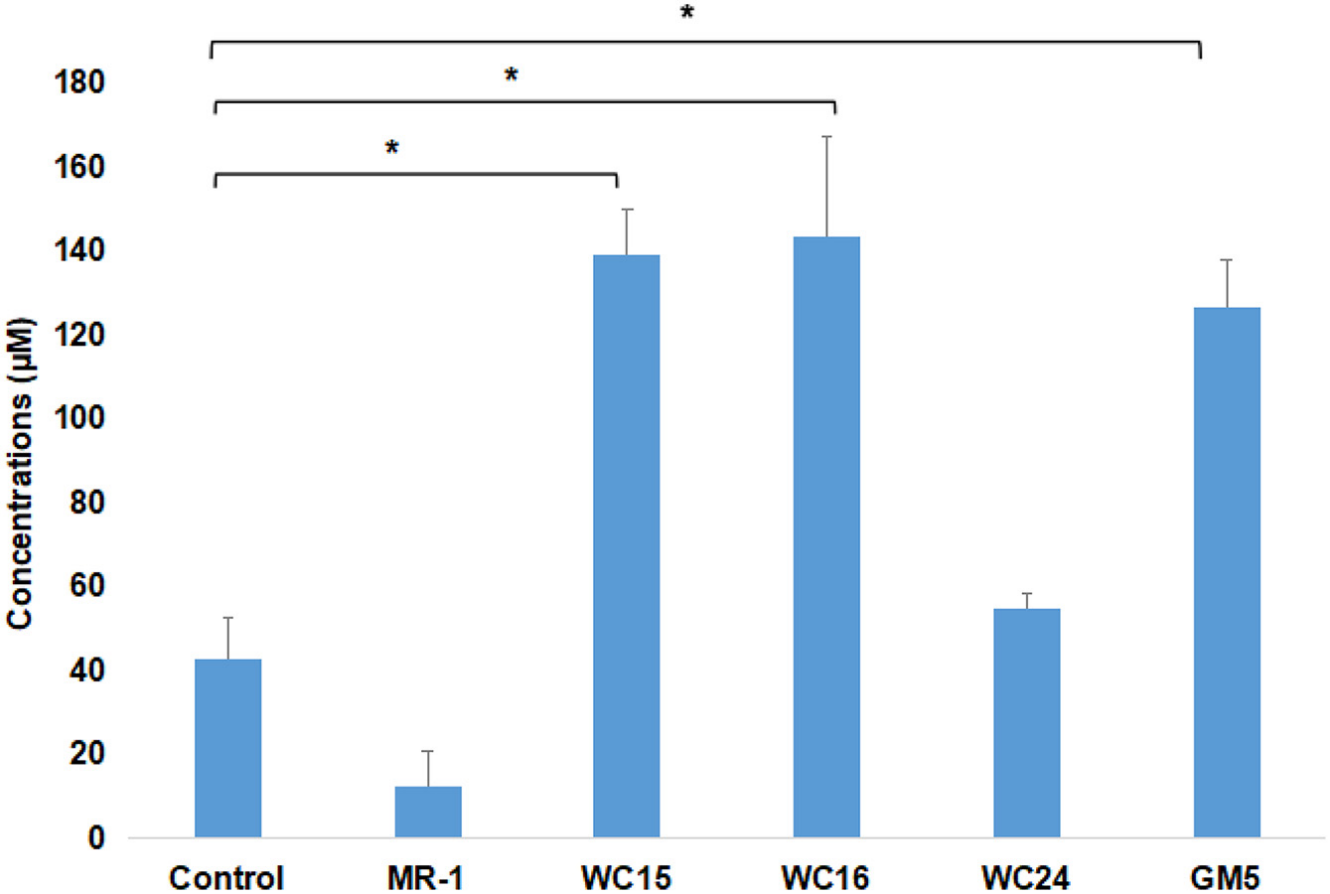
Quantification of IAA production by each strain supplemented with L-tryptophan. Control: not infected with any microbial strain, MR-1: inoculated with *Shewanella oneidensis* MR-1. WC15: *Rhizobium* sp., WC16: *Sphingomonas* sp., WC24: *Methylobacterium* sp., and GM5: *Bradyrhizobium* sp. *P<0.05.

The studied isolates - *Bradyrhizobium* sp. (GM5), *Rhizobium* sp. (WC15), *Sphingomonas* sp. (WC16), and *Methylobacterium* sp. (WC24) - were screened for their ability to produce IAA in a culture media supplemented with L-tryptophan. The IAA production indicated that *Sphingomonas* sp. produced the highest amount of IAA (143.3 ± 24.92 μM), followed by *Rhizobium* sp. (139.0 ± 11.87 μM), *Bradyrhizobium* sp. (126.8 ± 11.96 μM), and Methylobacterim sp. (56.7 ± 4.76 μM). The IAA concentrations were found to be significantly higher (*p* < 0.05%) in *Sphingomonas* sp. (2.36 fold), *Rhizobium* sp. (2.26 fold), and *Bradyrhizobium* sp. (1.97 fold) when compared to the control group (42.6 ± 10.12 μM). This confirmed that the selected strains produced IAA (Fig. 4). *S. oneidensis* MR-1 was used as a negative control in the present study because it lacked a homolog to the TnpA (tryptophan-indol lyase) enzyme that converts tryptophan to indol, which resulted in a low level of IAA (12.7 ± 8.01 μM) in the MR-1 strain. The MR-1 utilizes tryptophan-based signaling molecules in Biofilm preparation [28]. A statistical analysis using a one-way ANOVA test revealed a significant difference between the control and the treated samples (*p value < 0.05).

### Symbiotic Roperties of the Isolated Bacterial Strain

**Fig. 5:**
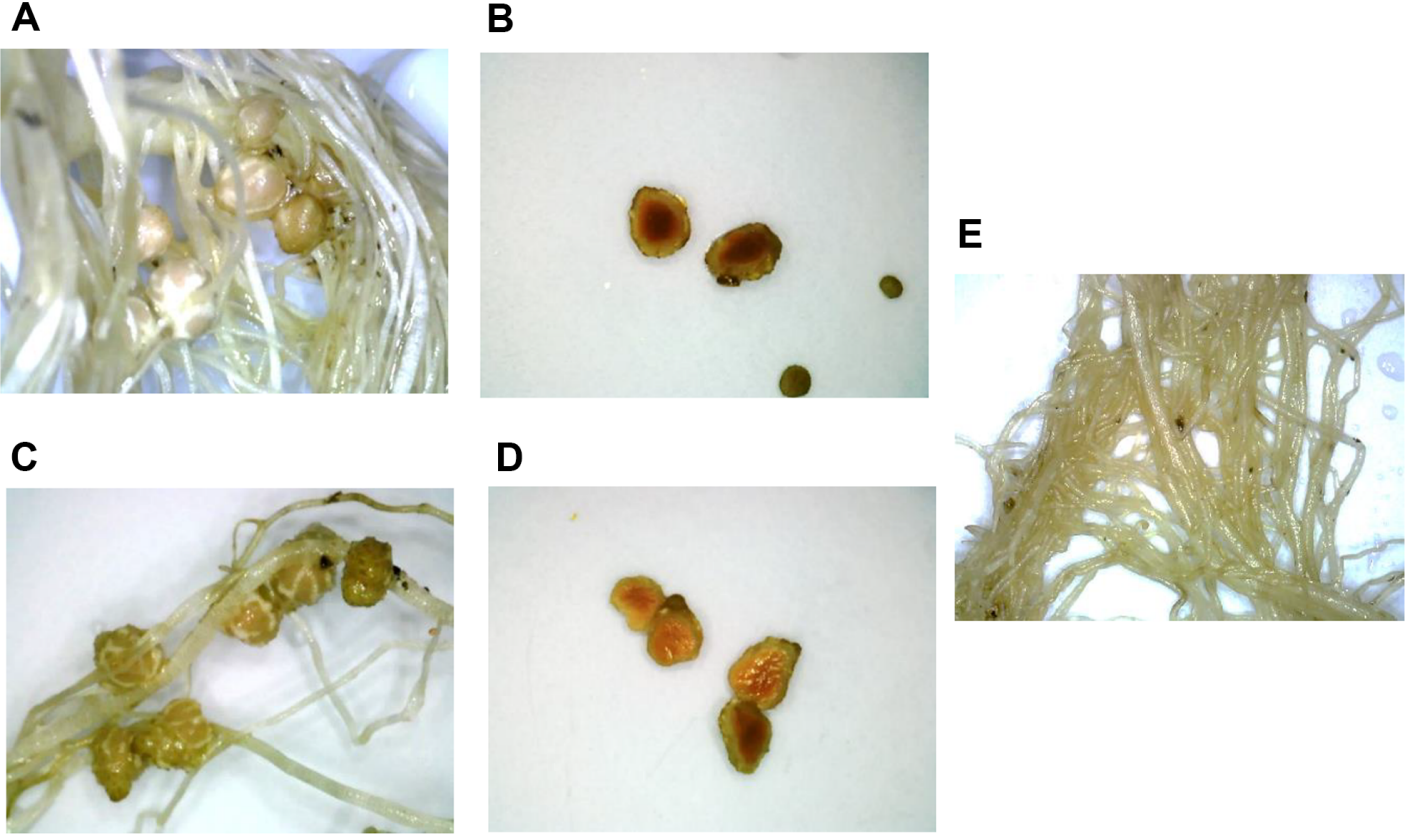
Root nodule morphology and cross-section of nodules from (A and B) GM5 (*Bradyrhizobium* sp.), (C and D) WC24 (*Methylobacterium* sp.) inoculated, and (E) Control.

The surface-sterilized seeds of *Trifolium repens*, *Glycine max*, and *Phaseolus vulgaris* were germinated on a 1%water-agar media for 2-3 days, transferred to the pots at one seed per pot, and allowed to grow for approximately 40 days under controlled conditions. On the third day, the bacterial isolates WC15 (from *T. repens*), WC16 (from *T. repens*), WC24 (from *T. repens*), GM5 (from *G. max*) were inoculated into each pot. The formation of pink nodules was observed on the roots of *Glycine max* inoculated with WC24 and GM5, which suggested that these strains were effective potentially in nitrogen fixation in *Glycine max*. In contrast, root nodules were not observed in the experimental pots of *T. repens* and *P. vulgaris*, inoculated with the isolated strains of WC15, WC16, WC24, and GM5, suggesting that they were not microsymbionts for these particular legumes. These finding indicated that the *Methylobacterium* strain (WC24), isolated from the root nodules of *T. repens* was able to induce nodulation in *G. max*. The effect of the nodulation on the dry weight of the root was evaluated. The plant roots inoculated with WC24 weighted the most (0.038 ± 0.017 g), followed by GM5 (0.035 ± 0.002 g). As shown in Table 4, the number of nodules and dry nodule weight (g) per plant was higher in the plants inoculated with GM5 than those inoculated with the WC24. The cross-section of the root nodules collected from the *G. max* that were inoculated with the isolated bacteria (GM5 and WC24) indicated a potential symbiotic hemoglobin (leghemoglobin), observed by a red color (Fig. 5).

### Absolute Expression of the *nifH* Gene Using qRT-PCR

**Fig. 6:**
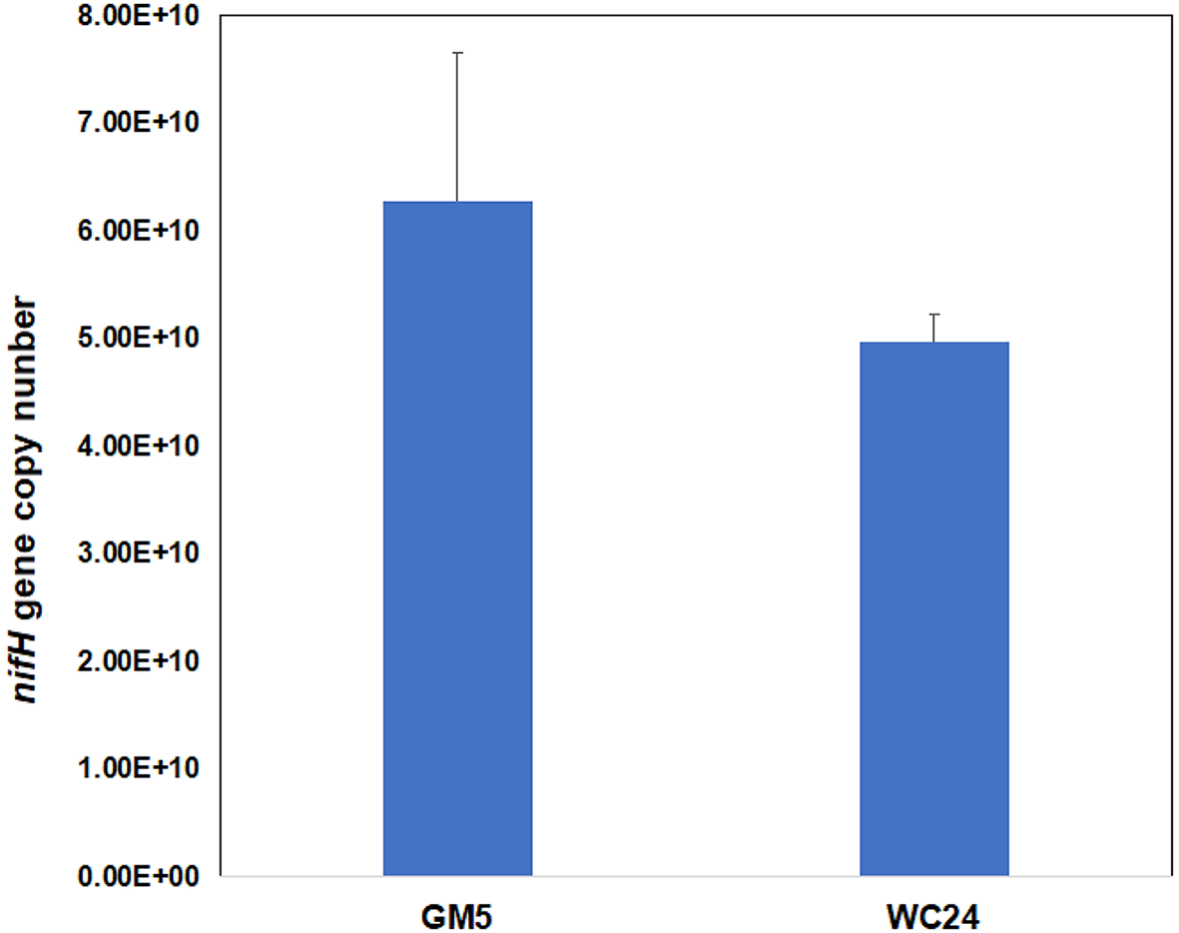
Absolute copy numbers of the *nifH* gene in the *Glycine max* nodules induced by strains GM5 (*Bradyrhizobium* sp.) and WC24 (*Methylobacterium* sp.).

The *nifH* gene is commonly used as a marker gene to track N_2_ fixation in plants. The expression of the *nifH* gene in both strains (WC24 and GM) that showed nodulation was quantified using qRT-PCR. The GM5 (*Bradyrhizobium* sp.) showed a higher expression of the *nifH* gene, measured at 6.27 × 10^10^ ± 1.39 × 10^10^ gene copy number per 1 g of *G. max* root nodules. The WC24 (*Methylobacterium* sp.) produced 4.97 × 10^10^ ± 2.53 × 10^9^ gene copy number per 1g of *G. max* root nodules (Fig. 6). A higher expression of the *nifH* gene is usually related to a higher N_2_ fixation ability of plants. The existence and expression of the *nifH* gene in both isolates indicated the N_2_ fixation ability of the *G. max*, with both the host plant-originated strain GM5 and the non-host plant-originated strain WC24.

## Discussion

The N_2_-fixing ability of rhizobia is essential for adding nitrogen to the soil, which in turn increases the soil fertility and crop productivity. The association of rhizobia and legumes is highly specific, and each rhizobial strain establishes a symbiotic relationship with a specific legume plant. The host specificity of rhizobia is observed at both the genus and species levels [6]. A certain level of mismatch between the two symbiotic partners is tolerated for the development of symbiosis. The 16S rRNA gene sequencing and phylogeny were successfully used to identify the rhizobial symbionts collected from the root nodules of five different leguminous plants (*Trifolium repens*, *Wisteria floribunda*, *Pisum sativum*, *Vigna radiate*, and *Glycine max*). The tested rhizobia - WC15, WC16, WC24, and GM5 - displayed the highest percentage of similarity to the genera *Rhizobium*, *Sphingomonas*, *Methylobacterium*, and *Bradyrhizobium*, respectively. Previous studies have reported the potential of the full-length 16S rRNA gene sequence to accurately identify bacterial species with a high taxonomic resolution [27, 31-34].

The phylogenetic relationships among the bacterial isolates were tested based on their symbiotic genes (*nifH* and *nodC*). The neighbor-joining phylogenies of the *nifH* and *nodC* genes were consistent with the 16S rRNA gene-based phylogeny, which suggested that these symbiotic genes were coevolved [27, 32, 35-36]. Incongruencies in the *nifH* and *nodC* phylogenies have been previously reported [37, 38], and a higher sequence similarity of the *nodC* gene was reported in *Bradyrhizobium* and *Mesorhizobium* spp. [39]. This supported the nodulation of *G. max* in this study, which was inoculated by a non-host plant-originated bacterium.

The present study demonstrated that most of the bacterial isolates were able to produce significant amounts of IAA in the presence of tryptophan. In in vivo conditions, the isolated rhizobia utilized L-Tryptophan as a substrate for the synthesis of IAA (auxin), which controls the various physiological processes in plants [18, 19, 21, 40]. Previous reports have confirmed that IAA is produced by different symbiotic and non-symbiotic nitrogen-fixing bacteria [20, 41, 42].

A cross inoculation experiment evaluated the nodulation and N_2_ fixation ability in relation to the isolated strains: *Rhizobium* (WC15), *Sphingomonas* (WC16), *Methylobacterium* (WC24), and *Bradyrhizobium* (GM5). These were used as inoculants for the three different leguminous plant species, *T. repens*, *G. max*, and *P. vulgaris*. The finding showed that the *Methylobacterium* strain (WC24) from *T. repens* nodules induced nodulation in the *G. max* roots, and produced a significant number of nodules. Root nodules were observed in the *G. max* infected with *Bradyrhizobium* (GM5). The *Bradyrhizobium* species - particularly *B. elkanii*, *B. japonicum*, *B. diazoefficiens*, *B. liaoningense*, and *B. yuanmingense* - are the native nodulating species of *G. max*, whereas *Methylobacterium* is not a native nodulating bacterium for *G. max* [43, 44]. The cross-section of the root nodules collected from the infected *G. max* indicated the presence of leghemoglobin (hemeprotein). Leghemoglobin is a symbiotic hemoglobin protein that creates the anaerobic conditions inside the nodules which is necessary for the effective symbiotic nitrogen fixation [11, 12, 45].

A previous report showed that the expression of the *nifH* gene was positively correlated with a higher nitrogen fixation [24, 46]. The expression of the *nifH* gene in the isolated strains of WC24 and GM5 indicated their high N_2_ fixation ability in the *G. max* plant. This study’s findings suggested that there was a symbiotic compatibility between *G. max* and the *Methylobacterium* strain from *T. repens* nodules. The nodulation capacity and nitrogen-fixing ability of the isolated bacteria indicated the possibility of using these eco-friendly microbial agents as biofertilizers. While these findings are a beginning to developing a biofertilizer, the nitrogen fixation efficiency of the infected *G. max* needs to be evaluated under field conditions and a greater number of isolated strains still need to be evaluated with other leguminous plants to truly develop an effective universal biofertilizer.

## Figures and Tables

**Table 1:** Plant collection sites for collecting root nodules.

Host legume plant	Collection site
*Trifolium repens*	Jeonbuk National University, Jeonju, South Korea
*Wisteria floribunda*	Jeonbuk National University, Jeonju, South Korea
*Pisum sativum*	Wild field, Jeonju
*Vigna radiate*	Wild field, Jeonju
*Glycine max*	Wild field, Nonsan

**Table 2:** Primer sequences used in PCR and RT-qPCR analysis.

Target gene	Primer name	Primer sequence
16S rRNA	27_F	5'-AGAGTTTGATCMTGGCTCAG-3'
	1492_R	5'-TACGGYTACCTTGTTACGACTT-3'
*nifH*	DVV_F	5'-ATIGCRAAICCICCRCAIACIACRTC-3’
	IGK3_F	5'-GCIWTHTAYGGIAARGGIGGIATHGGIAA-3’
	Pol_F	5'-TGCGAYCCSAARGCBGACTC-3’
	Pol_R	5'-ATSGCCATCATYTCRCCGGA-3'
*nodC*	*NodC*_F 540	5'-TGATYGAYATGGARTAYTGGCT-3’
	*NodC*_R 1160	5'-CGYGACARCCARTCGCTRTTG-3'
GAPDH	GAP_F	ACACCCACTCCTCCACCTTTG
	GAP_R	TCCACCACCCTGTTGCTGTAG

**Table 3:** The percentage similarity of bacterial isolates with the closest species in the GenBank based on candidate gene sequences.

Query	Scientific Name	Max Score	Query Cover	E value	Per. ident	Accession
Similarity-based on the 16S rRNA gene						
WC15	*Rhizobium* sp.	1897	99%	0	98.17%	MN049731.1
	*Rhizobium* sp.	1897	99%	0	98.17%	OQ865644.1
	*Rhizobium* sp.	1897	99%	0	98.17%	KC236648.1
WC16	*Sphingomonas* sp.	2073	99%	0	98.08%	MN989151.1
	*Sphingomonas* sp.	2073	99%	0	98.08%	MT749849.1
	*Sphingomonas* sp.	2050	98%	0	98.06%	FR696369.1
WC24	*Methylobacterium komagatae*	2017	99%	0	99.37%	AB698710.1
	*Methylobacterium* sp.	1986	99%	0	98.92%	MN508464.1
	*Methylobacterium* sp.	1980	99%	0	98.75%	MN982827.1
GM5	*Bradyrhizobium* sp.	1991	100%	0	99.91%	KY941256.1
	*Bradyrhizobium* sp.	1986	100%	0	99.82%	KY941249.1
	*Bradyrhizobium elkanii*	1969	100%	0	99.54%	MN338958.1
Similarity based on the *nifH* gene						
WC15	*Rhizobium* sp.	630	100%	5.00E-179	100.00%	KX394363.1
	*Rhizobium* sp.	512	99%	2.00E-143	93.82%	CP021375.1
	*Rhizobium* sp.	448	99%	5.00E-124	90.53%	KR075967.1
WC16	*Sphingomonas azotifigens*	571	100%	2.00E-161	100.00%	AB217474.1
	*Sphingomonas* sp.	542	98%	2.00E-152	98.69%	FJ455053.2
	*Sphingomonas* sp.	538	98%	2.00E-151	98.68%	FJ455037.2
WC24	*Methylobacterium aquaticum*	616	100%	4.00E-175	100.00%	AB935112.1
	*Methylobacterium aquaticum*	616	100%	4.00E-175	100.00%	AB598550.1
	*Methylobacterium aquaticum*	616	100%	4.00E-175	100.00%	AP014705.1
GM5	*Bradyrhizobium elkanii*	1279	100%	0	100.00%	AP013103.1
	*Bradyrhizobium yuanmingense*	1279	100%	0	100.00%	LC461082.1
	*Bradyrhizobium* sp.	1279	100%	0	100.00%	MF140389.1
Similarity-based on the *nodC* gene						
GM5	*Bradyrhizobium elkanii*	1090	100%	0	100.00%	AP013103.1
	*Bradyrhizobium elkanii*	1090	100%	0	100.00%	KY607996.1
	*Bradyrhizobium elkanii*	1090	100%	0	100.00%	CP126007.1

**Table 4:** Effect of isolated strains on root growth and nodulation.

	Dry root weight (g)	Dry nodule weight (g)	Number of nodules
Control	0.029 + 0.002		-
MR1	0.031 + 0.004		-
GM5	0.035 + 0.002	0.007 + 0.004	11
WC24	0.038 + 0.017	0.004 + 0.003	6
WC15	0.026 + 0.004		-
WC16	0.030 + 0.007		-
